# Applications of Transcriptomics in the Research of Antibody-Mediated Rejection in Kidney Transplantation: Progress and Perspectives

**DOI:** 10.1080/15476278.2022.2131357

**Published:** 2022-10-19

**Authors:** Hsuan Yeh

**Affiliations:** Division of Renal-Electrolyte, School of Medicine, University of Pittsburgh, Pittsburgh, Pennsylvania, USA

**Keywords:** Kidney transplantation, antibody-mediated rejection, molecular diagnostics, microarray, transcriptome analysis, single-cell RNA sequencing, precision medicine

## Abstract

Antibody-mediated rejection (ABMR) is the major cause of chronic allograft dysfunction and loss in kidney transplantation. The immunological mechanisms of ABMR that have been featured in the latest studies indicate a highly complex interplay between various immune and nonimmune cell types. Clinical diagnostic standards have long been criticized for being arbitrary and the lack of accuracy. Transcriptomic approaches, including microarray and RNA sequencing of allograft biopsies, enable the identification of differential gene expression and the continuous improvement of diagnostics. Given that conventional bulk transcriptomic approaches only reflect the average gene expression but not the status at the single-cell level, thereby ignoring the heterogeneity of the transcriptome across individual cells, single-cell RNA sequencing is rising as a powerful tool to provide a high-resolution transcriptome map of immune cells, which allows the elucidation of the pathogenesis and may facilitate the development of novel strategies for clinical treatment of ABMR.

## Introduction

Allograft kidney transplantation has become a treatment of choice for patients with end-stage renal disease (ESRD) which has less impact on patients’ quality of life as compared with dialysis.^1^ Following the introduction of modern immunosuppressive regimens, the outcomes of kidney transplant recipients have been improving. However, chronic allograft rejection remains a knotty clinical issue despite the advances in immunosuppressants.^2^ Antibody-mediated rejection (ABMR) is reported to be the leading cause of chronic allograft rejection.^3,4^ Over the years, the weakness of diagnostic standards and the complexity of the immunological pathogenesis of ABMR have affected patient care and hindered the successful development of novel therapeutic strategies.

The major focus of this article is to review the advances in transcriptomic approaches including microarray, RNA sequencing (RNA-seq), and the rapidly emerging single-cell transcriptome analysis, with emphasis on their applications to the ABMR study. I attempt to provide a comprehensive overview of the insights and the opportunities that these techniques provide to improve the diagnosis and treatment of ABMR.

## Immunological mechanisms of ABMR

The allograft rejection starts from the recognition of alloantigens by recipient T cells. Allorecognition can be divided into direct, indirect, and semi-direct types.^5^ In direct allorecognition, the recipient T cells recognize the alloantigens presented by donor antigen-presenting cells (APCs). In indirect allorecognition, the alloantigens are processed into peptides by the recipient APCs and presented to the recipient T cells. The activated CD4 T cells help activate CD8 T cells to differentiate into cytotoxic effectors.^6^ In semi-direct allorecognition, recipient APCs acquire donor anti-human leukocyte antigen (HLA) molecules that present peptides directly to recipient T cells.^7^ In indirect allorecognition, T cells differentiate into T follicular helper T (Tfh) cells.^8^ The interaction between Tfh cells and B cells requires the signals of co-stimulatory and co-inhibitory molecules, and cytokines.^9^

After being activated by antigens, some of the B cells differentiate into short-lived plasma cells (SLPCs) that secrete antibodies, while some other B cells migrate to germinal centers and become long-lived plasma cells (LLPCs) or memory B cells with the aid of Tfh cells.^10,11^ Both LLPCs and memory B cells account for the donor-specific antibody (DSA) production; the quiescent memory B cells rapidly differentiate into SLPCs upon alloantigen re-exposure and account for the generation of de novo DSA, while the LLPCs constitutively secrete antibodies and produce long-term circulating DSA but do not react upon alloantigen re-exposure.^12^ The affinity maturation of the memory B cells in the germinal centers during the primary response involves mutations in the antigen combining site. This somatic hypermutation-based mechanism may account for the unpredictable nature of DSA in terms of antigen specificity and the frequent failure of optimal HLA matching by using serum alone.^12^

The formation of DSA results in three following consequences: complement-dependent cytotoxicity, antibody-dependent cellular toxicity, and direct endothelial injury.^13^ Upon the binding of DSA to alloantigen, the classical complement pathway is activated, which produces anaphylatoxins including C3a and C5a, recruits inflammatory cells, and ultimately leads to tissue injury.^14^ During this process, C4d is produced as a degradation product that binds to the endothelial basement membrane and appears as an in situ marker of complement activation in renal allografts.^15^ The Fc receptor binding to the Fc of innate immune cells including macrophages and natural killer (NK) cells is responsible for antibody-dependent cellular toxicity and leads to degranulation, cell lysis, and phagocytosis.^14^ The direct binding of the antigens on allograft endothelial cells also causes endothelial activation and proliferation. Instead of being a passive victim, the endothelial cells also participate in the pathogenesis of rejection including leukocyte adhesion and recruitment, lymphocyte activation and differentiation, as well as the secretion of cytokines and chemokines after activation.^16^

## Current diagnosis, treatment, and clinical challenges of ABMR

ABMR was first identified based on a very high correlation found between the occurrence of antibodies reactive to graft antigens and histologic evidence of microvascular lesions.^17^ Later on, it was found to be associated with classical complement activation based on the intense deposition of complement fragments C4d and C3d in peritubular capillaries shown by immunostaining.^18^

The diagnosis of ABMR in the current clinical practice primarily depends on the Banff classification. The Banff Classification of Kidney Allograft Pathology was initially established in August 1991 by a group of pathologists and clinicians in Banff, Alberta, Canada. Diagnostic criteria for acute/active ABMR were first added to the Banff classification after the 2001 conference.^19^ Since then, Banff conferences have been held every 2 years worldwide, and several major revisions and modifications have been made to improve the diagnostic and prognostic values. According to the most recent Banff 2019 Kidney Meeting Report,^20^ all three of the criteria listed below must be met for diagnosis: (1) histological evidence of allograft injury via microvascular inflammation (MVI), intimal or transmural arteritis, acute thrombotic microangiopathy, or acute tubular injury in the absence of any other apparent cause; (2) histological evidence of antibody-endothelial interactions either by C4d deposition, at least moderate MVI, or increased expression of gene transcripts/classifiers in the biopsy tissue that have been validated to be strongly associated with ABMR; and (3) the presence of circulating DSA, predominantly HLA antibody. C4d staining or expression of validated transcripts/classifiers as noted in criterion (2) may substitute for DSA, and biopsies meeting criterion 1 but not criterion 2 with current or prior evidence, but not remote DSA, may be stated as showing chronic ABMR.

In retrospect, the Banff classification was once arbitrated by the recognition of C4d deposition in peritubular capillaries. C4d positive staining in peritubular capillaries was the only indicator of antibody interaction with vascular endothelium in criterion (2) and must be met to make the diagnosis of ABMR according to the 2007 Banff classification.^21^ This has been shown to be problematic since many cases with features of chronic antibody-mediated rejection were observed to be C4d-negative, which suggests that C4d may be specific but not sensitive^22^; on the other hand, C4d staining may be positive in the absence of clinically significant ABMR.^23^ As a result, one major revision made in the 2013 Banff classification was to include C4d-negative ABMR, embracing other features including MVI and validated molecular markers as an alternative assessment for criterion (2).^24^ Further, the Banff working group specified the molecular markers as the expression of gene transcripts/classifiers associated with ABMR as a major update from the 2015 to 2017 Banff classification.^25,26^ The revised Banff classification was found to outperform previous versions in terms of ABMR recognition and prediction of graft loss,^27^ and such advances are largely aided by the results yielded with some of the techniques listed in the present article which will be described in detail in the next section.

Despite these major advances, the current Banff classification still carries several pitfalls, including the subjective quantitative scoring of histological lesions, uncertain pathogenesis for similar morphologies, and difficulty in determining the activity of inflammatory cells.^23^ In addition, the complexity of the participating cells and the immune crosstalk among them have created an arduous challenge in the development of effective treatments for ABMR. Common therapeutic strategies are based on the reduction of antibody titers using plasmapheresis, along with intravenous immune globulins (IVIG) plus immunosuppressants such as tacrolimus or mycophenolic acid.^6^ Novel agents including antibodies targeting B cells, plasma cells, and the complement system have featured in recent studies of ABMR. However, an updated systemic review and meta-analysis done by Wan et al.^28^ reported that rituximab showed little or no benefit when used in addition to the standard-of-care combination of plasmapheresis plus IVIG, and the efficacy of bortezomib and complement inhibitors for the treatment of ABMR remained unclear. Likewise, studies evaluating the beneficial effect of eculizumab, a humanized monoclonal antibody that binds to the human C5 complement protein, have reported mixed results in treating ABMR.^29–31^ Therefore, there is a constant unmet need for increasing the diagnostic precision in ABMR and in-depth knowledge of the interplay between the immune and nonimmune cells in the onset and progression of ABMR.

## Improving the diagnosis of ABMR: transcriptome analysis

The unsolved inconsistency in the conventional diagnostic system has called for the discovery and validation of molecular diagnostic tools in ABMR assessment. In transplantation, an allograft meets the graft-infiltrating cells containing genomes from both the donor and the recipient, which not only contributes to the variability of outcomes but also confers a unique genetic perspective on the approach to the illness.^32^ This section will cover the transcriptomic techniques that have been utilized to improve the diagnostic accuracy of ABMR including reverse transcription quantitative real-time polymerase chain reaction (RT-qPCR), microarray, and RNA-seq, and summarize major research corresponding to each technique.

Since the late 1990s, RT-qPCR has shown promise to explore differential gene expression (DGE) in various diseases. The principal steps of RT-qPCR include RNA isolation, generating complementary DNA (cDNA) with reverse transcriptase, and PCR amplification of cDNA with primers for specific genes of interest, in which DNA molecules labeled with fluorescent probes can be quantified by monitoring the signals.^33^ In renal allograft rejection, RT-qPCR has revealed elevation of multiple immune activation transcripts in allografts, including expression of interleukin (IL)-2, IL-7, IL-10, IL-15, Fas ligand, perforin, and granzyme B in acute renal allograft rejection,^34,35^ as well as transforming growth factor beta 1 (TGF-β1) in chronic rejection.^35^

First introduced by Brown et al.^36^ at Stanford University in 1995, microarray emerged as another powerful tool in molecular studies of transplant biopsies. In a typical microarray experiment, the mRNA extracted from tissues of interest is first converted into cDNA and tagged with fluorescent probes. The cDNA samples are then hybridized with numerous transcript sequences printed at a high density on a microscope slide. Following the hybridization, the microarray slide is then scanned to measure the fluorescence, which reveals the expression of each gene printed on the slide. Thus, microarray has the distinguished capability of monitoring the expression of many genes in parallel, and it has also been widely applied to reveal DGE in transplant rejection and, more specifically, ABMR.^37–44^

Some studies have compared the quantification of mRNA gene expression in renal allograft biopsies by RT-qPCR with the results yielded by microarray. One study reported that RT-qPCR and microarray gave similar results in abnormal kidneys and were both competent to detect the relevant changes in rejection.^45^ In contrast, a meta-analysis of the kidney microarray dataset investigating cytokine gene detection and the correlation with RT-qPCR results showed that microarray failed to detect a majority of cytokine-related genes, which are generally expressed at low abundance.^46^ Hence, it is worth attention that microarray, despite being high throughput, is limited by the range of detection owing to both background and saturation of signals, and could lead to false-negative results especially in detecting changes in the expression of genes of low abundance. Moreover, one major drawback shared by RT-qPCR and microarray is that both methods rely upon existing knowledge about genome sequence. RNA-Seq, emerging on the advent of next-generation sequencing (NGS) technology, can address these limitations.

RNA-Seq has clear advantages over the existing approaches and revolutionized the manner of transcriptome analysis.^47^ In a typical RNA-seq experiment, a population of RNA is extracted from samples, converted into cDNA, made into an adaptor-ligated sequencing library, and then sequenced to a depth of 10–30 million reads per sample.^48^ Exploiting the advances in computational methods, the resulting sequencing reads are either aligned to a reference genome or assembled de novo without the genomic sequence to produce a genome-scale transcription map for analyzing DGE.^47,48^ RNA-seq has been increasingly utilized in the investigation of different types of renal allograft rejection, including ABMR, albeit currently available studies are relatively limited compared to microarray method. One study performing RNA-seq on biopsy-paired peripheral blood samples from patient cohorts with different types of rejection identified 102 genes with enrichment in the regulation of endoplasmic reticulum stress, adaptive immunity, and Ig class-switching to be associated with ABMR, including the *SIGLEC17P* pseudogene and the related coding genes,^49^ of which the expression is almost exclusively in NK cells.^50^ Dooley et al.^51^ performed RNA-seq with human urine samples matched to TCMR, ABMR, as well as non-rejection biopsies and identified three novel mRNAs (*ITM2A, SLAMF6*, and *IKZF3*) of which the expressions were significantly higher in urine matched to TCMR or ABMR than in the non-rejection biopsies.

In terms of the translation of these transcriptomic techniques into clinical diagnostics, one known example is the work done by the Edmonton group. To facilitate the determination of the molecular phenotype of renal allograft biopsies using microarray, Mueller et al.^39^ in Edmonton, Alberta, first created pathogenesis-based transcript sets (PBTs) to reflect the biological processes in the alloimmune response. The study showed no absolute specificity of individual molecules or PBTs for rejection but revealed quantitative specificity, including interferon-gamma (IFN-γ) for rejection, T cell and macrophage transcripts for T cell-mediated rejection (TCMR), as well as endothelial and NK cell transcripts for ABMR.^39^ Based on the hypothesis that alloantibody acting on the microcirculation could be a sensitive indicator of ABMR, Sis et al.^40^ identified 119 endothelial-associated transcripts (ENDATs) from literature and studied their expression by microarray in 173 renal allograft biopsies; ENDAT expression was correlated with histopathologic lesions of ABMR but not TCMR, and high ENDAT expression in patients with alloantibody could serve as an indicator of active ABMR and poor graft outcome. To distinguish between ABMR and TCMR with molecular tests, the Edmonton group performed microarray on renal allograft biopsies to develop classifiers that assigned ABMR scores to each biopsy that had been assigned diagnoses including C4d-negative ABMR based on histology and donor-specific HLA antibody.^41^ The author group then conducted the prospective INTERCOM study (NCT01299168) in biopsies obtained from six centers: Baltimore, Barcelona, Edmonton, Hannover, Manchester, and Minneapolis to validate the ABMR score, which showed that the score was more strongly associated with allograft failure than conventional assessments and could predict early progression to failure.^42^ Further, the author group developed the Molecular Microscope Diagnostic System (MMDx) using machine learning-derived classifier algorithms,^43^ which showed better agreement with clinical judgment than histology did in an extended survey of the INTERCOM study (INTERCOMEX).^44^ The serial studies demonstrated the feasibility of molecular assessment in biopsy interpretation.

The measurement of ENDAT and other immune activation transcripts in allograft has also been used to evaluate the therapeutic effects of novel immunosuppressive regimens on ABMR. Kulkarni et al.^31^ assessed the efficacy of eculizumab therapy for chronic ABMR with ENDAT confirmed by RT-qPCR in a pilot randomized controlled trial. Apart from this study, most previous animal model studies and clinical trials of immunosuppressive treatments for ABMR used renal function, serum C4d, or DSA levels for outcome monitoring instead of DGE. A possible explanation is that the patients who met the diagnostic criteria with positive results for C4d and DSA might not be tested for ENDAT and would not be monitored with ENDAT as parameters in the follow-up studies. Nevertheless, since gene classifiers/transcripts have already been included in the Banff classification and their significance in differential diagnosis has been widely recognized, the measurement of ENDAT and other immune activation transcripts in allograft still has great potential to increasingly serve as biological markers for treatment response.

The advances in transcriptome profiling techniques have facilitated the refinement of the diagnostic Banff Classification and have shown their great potential to become useful adjuncts to histopathology-based diagnostics. However, there are still unaddressed issues related to improving clinical care and patient outcomes. Regarding improving diagnosis, the microarray-based MMDx, the most established model of applications of transcriptomics in ABMR, has received some critiques from pathologists specifying its questionable statistical measures of variability for classifier scores used for MMDx assignment, the inability to differentiate overlapping phenotypes such as non-rejection inflammation, thresholding and sampling problems arising from the various degrees of inflammation within a tissue sample, and the interlaboratory variability of microarray-based assays.^52^ Regarding progress in therapeutic strategies, the treatment responses to conventional immunosuppressive therapies vary widely and the outcomes are generally suboptimal. Such treatment hurdles may arise from the complex immune mechanisms and the heterogeneity of the cell types that are involved in the development of ABMR, including not only the immune cells but also the resident cells in the allograft.^15,53^ Moreover, the extremely dynamic nature of the immunological response, as well as the ever-changing surface expression of surface markers of immune cells, have also been reported as causes of resistance to treatments.^13,54,55^

The transcriptomic approaches described in this section analyze bulk samples, which only reflect the average gene expression but not the status of individual cells and are unable to distinguish the gene expression profile of donors from recipients in a mixed cell population.^56,57^ To make a breakthrough in either improving diagnosis or treatment, it is important to address the cellular heterogeneity and to dissect the behaviors of individual cells, so as to identify the cell populations that play a decisive role in the response or lack of response to certain treatments and to determine whether patients, of which clinical and molecular phenotypes, at which time point of the disease course, may benefit from any given treatments. The advent of single-cell RNA sequencing (scRNA-seq) could address cellular heterogeneity and has largely improved our understanding of transcriptomics during cell–cell interaction.^58^ In the following two sections, I will touch on the basic principles of scRNA-seq, describe how it has been utilized to decipher the transcriptomes of various cell subtypes in studies of ABMR, and catalog the published data yielded with this technique.

## Single-cell transcriptome analysis in ABMR

**Figure 1.**
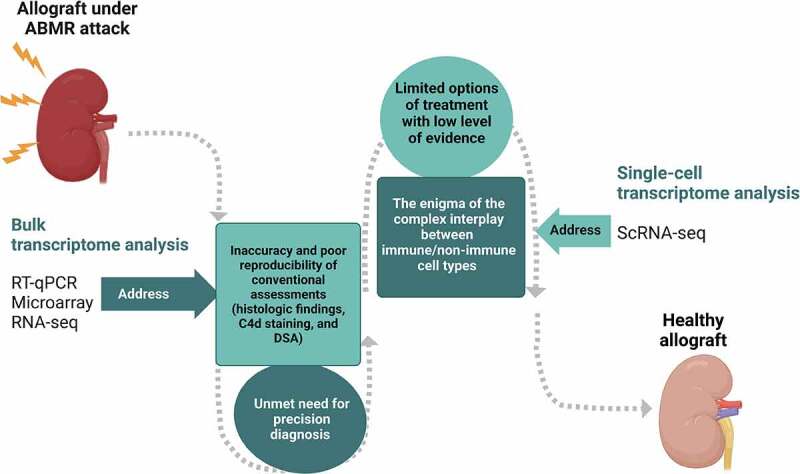
The potential clinical applications of transcriptomic approaches to improve graft outcomes in patients with antibody-mediated rejection in kidney transplantation. The figure was created with BioRender.com.

ScRNA-seq is a promising research tool that provides unprecedented resolution to examine cellular heterogeneity in tissues and organs in a high-throughput fashion,^59^ and it exhibits unique values in providing new insights into renal pathophysiology. There are more than 20 cell types with distinct spatial organization in kidneys,^60^ and the behaviors and gene expression in response to certain insults or pathological conditions, including ABMR, may differ between different compartments of the organ.^61^ ScRNA-seq not only detects specific changes of a known cell type but also redefines kidney cell types based on the transcriptome patterns.^53^ As a result, it can provide a new entry point for enhancing the knowledge of the immunological response behind the rejection and for developing the therapeutic strategy that has been limited by the complexity of the disease (Figure 1). In addition, its distinguished ability to present a dynamic and transient profile of the cell–cell interaction makes it a powerful tool to aid our understanding of immunologic mechanisms in renal allograft rejection.^57^

The technique encompasses the following principal steps. Briefly, single cells are isolated from a tissue sample by various methods including serial dilution, micromanipulation, microdissection, flow cytometry and cell sorting (FACS), magnetic-activated cell sorting (MACS), microfluidic platform, and droplet-associated methods.^62^ Next, isolated cells are lysed individually to allow the capture of RNA molecules. Poly T primers that bind to the mRNA poly A tails are used to capture the mRNA. The mRNA is then converted to cDNA and amplified by reverse transcription. The primers used in this step contain adaptor sequences and unique molecular identifiers (UMIs) to label the cellular origin. The cDNA is then amplified by polymerase chain reaction, pooled, and analyzed using library preparation techniques, sequencing platforms, and genomic alignment. Data analysis after sequencing usually comprises quality control, batch effect correction, normalization, data imputation, dimensionality adjustment, expression analysis, and cell subpopulation identification.^63^

There are some major challenges in sample preparation and cell isolation for biopsies obtained from patients. In certain areas, the distance between the clinics and laboratories may hamper the timely delivery of samples.^64^ As a result, tissues often but not always require special processing after collection in clinical scenarios. Cryopreservation and methanol fixation are the most used methods for tissue preservation, and currently available evidence shows that both methods do not significantly alter the integrity of nucleic acids or the transcriptome profiles.^65–67^ However, according to the observation and the comments by Wu and Humphrey,^68^ adult kidneys contain a relatively dense matrix and are difficult to be enzymatically digested, and there is indeed a chance that proteolytic dissociation becomes a stressor to transcriptomes and RNA integrity, although that has not been evidenced by comparative studies.

Single-cell isolation is the most critical step for obtaining transcriptome profiles from an individual cell. Each of the aforementioned methods that are commonly used to isolate single cells from tissues has its own strengths and flaws.^64^ Of them, droplet-associated methods and FACS are mostly used in currently available scRNA-seq studies in kidneys (Table 1).^53,57,76^ FACS has the strength of being able to sort cell populations with high specificity by evaluating multiple parameters at once but has downsides including being operator-dependent, need for specialized equipment, and more cell damage^64^; it also requires large starting volumes of cells (>10,000 cells) in suspension, which hinders its application to analyze limited tissue samples.^77^ In terms of droplet-based methods, the three most commercially available systems are Drop-Seq, InDrop, and 10X Genomics Chromium. Drop-Seq has been extensively used but has a low cell capture rate (<5%) and is poorly suitable for cell isolation from a small starting volume of renal biopsy,^68^ while the latter two systems have remarkably higher capture efficiency (>50%) and thus are much more capable of the analysis of human biopsies.^68,78,79^ 10X Genomics Chromium has become the most prevalent droplet-associated system in ABMR scRNA-seq studies based on the present review (Table 1).

**Table 1.** Summary of single-cell RNA sequencing studies on antibody-mediated rejection/chronic allograft rejection in kidney transplantation.

Reference	Method	Species	Specimen Type	Pinpointed Cell subtypes	Major Findings
Dangi *et al*.^70^	FACS	Mouse	Kidney	T cell, myeloid cell	Axl top gene augments intragraft differentiation of proinflammatory macrophages, which plays a major role in promoting intragraft myeloid cell and T cell differentiation.
Shen *et al*.^81^	FACS	Mouse	Kidney	Myeloid cell, T cell, B cell, neutrophil	The reduction in CD8+ T cells, B cells, neutrophils, and the increase in Ly6c^lo^Mrc1^+^ and Ly6c^lo^Ear2^+^ macrophages, may contribute significantly to the progress from acute rejection to chronic rejection.
Wu *et al*.^71^	InDrops (Droplet-based)	Human	Kidney	Monocyte, epithelial cell, endothelial cell, stromal cell	Monocytes form two subclusters: a nonclassical, proinflammatory CD16+ group (monocyte 1) and a classic CD16− group (monocyte 2). Monocyte 1 is strongly associated with allograft rejection and expresses dendritic cell maturation markers: *APOE, PDE3A, IGKC, LGMN*, and i*CD83*.
					Proximal tubule expresses proinflammatory cytokine genes, including *CXCL14*, and *IL32. Sox9*, a master regulator of proximal tubule repair after injury, is upregulated as well. Other renal tubular cells also amplify proinflammatory and profibrotic responses.
					Endothelial cells can be clustered into three separate cell states in rejection. The cluster that expresses the angiogenic program, as well as the cluster that shows increased expression of endoplasmic stress marker XBP1 and the cold shock gene *RBM3*, are associated with ABMR.
					Separate stromal clusters are identified. Cluster 1 (pericyte cell type) highly expresses *RSG5* and *CACNA1C*. Cluster 2 (fibroblast cell type) expresses *MoxD1*. Cluster 3 (myofibroblast cell type) uniquely expresses *COL8A1* and *COL12A1*.
					NKT cells form five subclasses, representing CD4+ T cells, CD8+ T cells, CTLs, regulatory T cells, and NK cells. A similar single-sample gene set enrichment pattern was observed in CD8+ T cells and CTLs, showing higher cytotoxic, antigen presentation, and proinflammatory activities, while NK cells exhibited weak immune activation states, except for encoding several granzyme molecules.
Liu *et al*.^72,^	10X Genomics (Droplet-based)	Human	Kidney	NKT cell, B cell, monocyte, myofibroblast	Two subtypes of memory B cells, activated and stationary states, are identified to be enriched in chronic kidney transplant rejection.
					Monocytes form a classic CD14+ group and a nonclassical CD16+ group. Monocyte activation pathways including MHC, INF-γ/β, and the inflammatory response were significantly higher in chronic rejection-derived monocytes.
					Myofibroblast is identified as a novel subpopulation. Chronic rejection allografts are characterized by increased numbers of immune cells and myofibroblasts.
Malone *et al*.^73^	10X Genomics (Droplet-based)	Human	Kidney	Macrophage, T cell	Recipient macrophages display inflammatory activation, whereas donor macrophages demonstrate antigen presentation and complement signaling.
					Recipient-origin T cells express cytotoxic and proinflammatory genes consistent with an effector cell phenotype, whereas donor-origin T cells express oxidative phosphorylation genes.
					Pathway analysis reveals proinflammatory pathways in recipient T cells, including IL-12 signaling through STAT4, IL-6 signaling, and downstream signaling in native CD8+ T cells, as well as the HIF-α transcription factor pathway.
Kong *et al*.^74^	GEXSCOPE (Microfluidics)	Human	Peripheral blood	T cell, B cell	The expression of *MT-ND6, CXCL8, NFKBIA*, and *NFKBIZ* genes are up-regulated in T and B cells and are associated with pro-inflammatory response and immune regulation.
Asano *et al*.^75^	FACS	Human	Kidney	B cell	Intrarenal class-switched B cells show an innate cell transcriptional state resembling mouse peritoneal B1 or B in cells. *AHNAK* gene is specifically upregulated in this type of cell.

FACS: flow cytometry and cell sorting; NKT: natural killer T; CTLs: cytotoxic T lymphocytes; NK: natural killer; IL: interleukin; STAT4: signal transducer and activator of transcription 4; HIF-α: hypoxia-inducible factor 1-alpha; Bin: B-innate

To date, published studies that utilize scRNA-seq to analyze ABMR, including both animal and human-based data, are relatively limited (Table 1). The cells of interest in most studies are isolated from the renal allograft biopsies or the peripheral blood, but the feasibility of scRNA-seq analysis of individual cells in urine samples matched to human kidney allograft biopsies has also been reported.^80^ The cell clusters that are identified to take part in ABMR can be generally classified into three types: cells in the innate immune system, cells in the adaptive immune system, as well as nonimmune populations including renal epithelial cells, endothelial cells, and stromal cells.

In 2018, Wu et al.^71^ published the first scRNA-seq analysis of a single human renal allograft biopsy. By generating data from single-cell transcriptomes from a healthy adult kidney and a single renal allograft diagnosed as chronic ABMR, they identified two subclusters of monocytes, a nonclassical FCGR3A(CD16)+ group and a classic CD16− group expressing dendritic cell (DC) maturation markers. CD16+ cells were strongly associated with allograft rejection and were found to highly express two receptors (SDC3 and ABCA1) and a panel of DC maturation markers, suggesting differentiation into DC in situ. Of the six distinct epithelial cell clusters identified, including podocytes, proximal tubules, the loop of Henle, distal tubules, principal cells, and intercalated cells, the proximal tubule particularly expressed proinflammatory cytokine genes, such as *CXCL14* and *IL32*. They also identified three clusters of donor endothelial cells and three clusters of stromal cells that were involved in the ABMR to various degrees. In 2020, Malone et al.^73^ used scRNA-seq to analyze human allograft biopsies and found that the recipient macrophages and donor T cells displayed inflammatory activation, and that donor macrophages persisted for years post-transplantation. Liu et al.^72^ collected scRNA-seq data from three healthy adult kidneys and two renal allograft biopsies of chronic renal transplant rejection; in addition to five subclasses of Natural killer T (NKT) cells, two subclasses of B cells, and two subclasses (classic and non-classical) of monocytes, they identified myofibroblasts, which expressed collagen-related genes, platelet-derived growth factor receptor (PDGFR)-related genes, and epithelial-to-mesenchymal transition (EMT)-related genes, as a novel subpopulation involved in the development of chronic renal allograft rejection. In another study, scRNA-seq of the peripheral blood from patients diagnosed with chronic ABMR and two healthy individuals identified four subtypes in T-cell subsets and two subtypes in B cell subsets; the expression of *MTND6, CXCL8, NFKBIA, NFKBIZ*, and other genes was up-regulated in both T and B cells and was associated with pro-inflammatory response, including the tumor necrosis factor (TNF), IL-17, and Toll-like receptor signaling pathways. Mitogen-activated protein kinase (MAPK) and nuclear factor kappa B (NF-kB) signaling pathways were also involved in the development of chronic ABMR.^74^ Finally, Asano et al.^75^ performed a scRNA-seq analysis focusing on the role of the B cell population in chronic ABMR. By comparing the Ig class-switch states and DGE between the human tonsillar and the intrarenal B cells, they reported that intrarenal B cells have a unique transcriptional state that resembles gene expression data of mouse B1 innate-like (Bin) cells acquired from The Immunological Genome Project (Immgen)^69^; several migration- and adhesion-related genes were highly expressed in this population.

ScRNA-seq analysis on animal models also provides an abundance of insights into the disease mechanism of ABMR. One study performing scRNA-seq on a murine kidney transplant model, in which BALB/c kidneys were transplanted into fully MHC-mismatched bilaterally nephrectomized C57BL/6 J recipients, demonstrated that the allograft-infiltrating myeloid cells followed a trajectory of differentiation from monocytes to proinflammatory macrophages and exhibited distinct interactions with kidney allograft parenchymal cells. Axl, a gene of the receptor tyrosine kinase family Tyro3/Axl/Mertk (TAM), was correlated with the differentiation.^70^ Another study performing scRNA-seq on CD45+ leukocytes in mouse renal allograft rejection model on days 7 and 15, respectively, showed that the proportion of proliferating and naïve CD8 + T cells, B cells, and neutrophils decreased, while the proportion of macrophages and DCs increased significantly, especially in Ly6c^lo^Mrc^1+^ and Ly6c^lo^Ear^2+^ macrophages during the progression from acute to chronic rejection.^81^

As described above, the currently available studies vary widely in the target species, the organ origins, and the matched diagnoses/experimental designs of the specimens, which might have led to mixed results in terms of the cell subtypes that were identified to be involved in the ABMR. However, some of these studies were in accordance with each other. A few of the aforementioned studies showed that the non-classical, CD16+ macrophages, had a major role in ABMR.^71,72^ Likewise, there was consistency among different studies that included the ligand and receptor (LR) pair analysis, reporting increased chemokine ligands production, such as CXCL8, CXCL12, or CXCL14 on various cell populations.^71,74^ Interestingly, more than one study pointed out that DGE not only mediated activation of immune cells but was enriched in the mitochondria oxidative stress and the endoplasmic reticulum (ER) stress,^71,73^ while the LR analysis in tolerated allograft found an increased Hsp90b1–Trl7 interaction,^70^ indicating that the cellular stress response was involved in the post-transplant ABMR, although its exact role and mechanism in the development of ABMR remains undetermined.

As with other techniques, scRNA-seq has its limitation. The basic steps of scRNA-seq require tissue samples to be dissociated into single-cell suspensions, which may change the transcriptome and proteome of cells. Tissue processing can also disrupt the spatial arrangement of cells in each organ, and the cell profile observed may not be expanded into other tissue environments.^82^ At a higher level of view, the disparity of experimental protocols across studies has not been systematically examined and would thus hamper the correct interpretation and further applications of the results. The optimization and standardization of the workflow of this technique, from the yield and the storage of tissue to data analysis, may help overcome the disadvantage and improve the consistency across studies. Currently, the relatively small sample number in most scRNA-seq studies, primarily due to the high cost of this technology, has also caused the divergence of the study goal. Thus, it is necessary to carefully examine the biological plausibility when drawing new conclusions from scRNA-seq data.^83^ Despite these limitations, scRNA-seq is developing rapidly and has shown great potential for discovering novel disease mechanisms and effective therapeutic strategies in clinical allograft rejection, including ABMR.

## Conclusion

The clinical management of ABMR is largely limited by not only the lack of an ideal diagnostic tool but also the diverse phenotypes and the elusive immunological mechanisms behind the disease. The transcriptome analysis including the microarray technique and RNA-seq enabled the identification of protein-coding and noncoding genes and the segregation of different rejection phenotypes. Such molecular diagnostics could be used in parallel with or even replace the conventional diagnostics based on Banff Classification to increase diagnostic accuracy and have shown promise to serve as biological markers for treatment response. To popularize the application of transcriptome analyses in response evaluation, more well-designed prospective clinical studies in which the baseline transcriptome profiles of patients are clearly documented for future comparisons are needed. ScRNA-seq is rising as a powerful tool to dissect cell–cell interactions, which sheds light on the complicated immunological interplay among various cell types in the allograft during the ABMR and identifies new cell populations that are involved in disease development. This would help explain the limitations of current treatments, as well as provide evidence for novel therapeutic strategies (Figure 1). Either animal or human-based scRNA-seq studies have been increasingly available. The shared results of scRNA-seq studies that have been conducted on ABMR reveal several panels of DGE associated with innate immunity, the chemotaxis of inflammatory cells, and the cellular stress response, which could be new targets for ABMR therapy. Although more studies under a uniform and well-established experimental protocol are awaited, it is hoped that the scRNA-seq technology can be widely translated into ABMR studies in humans, which will result in improved therapeutics for kidney transplant patients with chronic allograft dysfunction.
